# High-throughput biodiversity surveying sheds new light on the brightest of insect taxa

**DOI:** 10.1098/rspb.2024.2974

**Published:** 2025-05-14

**Authors:** Ela Iwaszkiewicz-Eggebrecht, Robert M. Goodsell, Bengt-Åke Bengsson, Marko Mutanen, Mårten Klinth, Laura J. A. van Dijk, Piotr Łukasik, Andreia Miraldo, Anders Andersson, Ayco Jerome Michel Tack, Tomas Roslin, Fredrik Ronquist

**Affiliations:** ^1^ Department of Bioinformatics and Genetics, Swedish Museum of Natural History, Stockholm, Sweden; ^2^ Lokegatan 3, Färjestaden, Sweden; ^3^ Department of Biology, University of Oulu, Oulu, Northern Ostrobothnia, Finland; ^4^ Station Linné, Färjestaden SE-38693, Sweden; ^5^ Faculty of Biology, Jagiellonian University, Krakow, Poland; ^6^ Department of Gene Technology, Science for Life Laboratory, KTH Royal Institute of Technology, Stockholm, Sweden; ^7^ Department of Ecology, Environment and Plant Sciences, Stockholm University, Stockholm, Sweden; ^8^ Department of Ecology, Swedish University of Agricultural Sciences, Uppsala, Sweden

**Keywords:** metabarcoding, high-throughput survey, DNA barcoding, Swedish Lepidoptera, biodiversity monitoring, species discovery

## Abstract

DNA metabarcoding of species-rich taxa is becoming a popular high-throughput method for biodiversity inventories. Unfortunately, its accuracy and efficiency remain unclear, as results mostly pertain to poorly known taxa in underexplored regions. This study evaluates what an extensive sampling effort combined with metabarcoding can tell us about the lepidopteran fauna of Sweden—one of the best-understood insect taxa in one of the most-surveyed countries of the world. We deployed 197 Malaise traps across Sweden for a year, generating 4749 bulk samples for metabarcoding, and compared the results to existing data sources. We detected more than half (1535) of the 2990 known Swedish lepidopteran species and 323 species not reported during the sampling period by other data providers. Full-length barcoding confirmed three new species for the country, substantial range extensions for two species and eight genetically distinct barcode variants potentially representing new species, one of which has since been described. Most new records represented small, inconspicuous species from poorly surveyed regions, highlighting components of the fauna overlooked by traditional surveying. These findings demonstrate that DNA metabarcoding is a highly efficient and accurate biodiversity sampling method, capable of yielding significant new discoveries even for the most well known of insect faunas.

## 1. Introduction

Nature enthusiasts invest millions of hours and unparalleled know-how in generating biological records. Such data form the basis of national checklists, trend analyses and ultimately Red Lists guiding conservation priorities [1,2]. Nonetheless, current attention by naturalists comes with biases in space, time and taxonomy [3]. As a key concern, analyses of such opportunistically recorded data are fraught with biases, as the underlying sampling effort will vary but is hard to characterize [4,5]. Larger, easier to spot or more charismatic species receive a disproportionate amount of attention compared with highly diverse but inconspicuous taxa, such as most insect species. Recently, standardized sampling techniques coupled with DNA sequencing have proven efficient in revealing ‘dark diversity’, by shedding new light on taxa that are small, hyperdiverse and taxonomically neglected [6–8]. To what extent the same methods may also brighten our view of the already well-lit parts of insect biodiversity remains poorly examined.

Of the methods available for accessing a comprehensive part of the local community, all have their drawbacks [9–11]. That a single method would sample *all* taxa with the same efficiency is an unrealistic expectation. In selecting a realistic solution, we need to account for at least two basic features of hyperdiverse taxa. First, given high diversity, we need to accumulate large numbers of individuals to characterize the many species in the community. Second, most species are rare, attesting to a need for intensive sampling in both space and time [12–14]. Both considerations call for the accumulation of large samples locally and comprehensive coverage in space and time. As a workable compromise between these requirements, many recent projects have drawn on the Malaise trap [15,16] as a wholesale approach to the sampling of flying insects [17–19].

With the sample in place, we need efficient means for taxonomic identification. Conventional identification by the low number of available taxonomic experts using morphological characteristics is inappropriate for samples containing millions of specimens. As a vivid example, insect samples from a single tropical forest took about a month to generate, but a decade to identify [20]. The sampling of around 1900 Swedish insect communities took 3 years to complete—but after two decades of intense work, less than 1% of the approximately 20 million specimens collected have been identified by expert taxonomists [21]. As a key alternative to morphology-based identification, DNA metabarcoding enables the rapid identification of mass samples of insects. By extracting and sequencing a short fragment of mitochondrial DNA—the barcode—from a bulk insect sample and then comparing the DNA sequence to reference databases, we may achieve an efficient snapshot of the biodiversity present in a particular place at a particular time [22]. These metabarcoding methods are readily applicable to samples of thousands to millions of insects.

By leveraging advances in bioinformatics and molecular techniques, the coupling of mass sampling of insects with high-throughput metabarcoding holds the potential to significantly enhance our ability to monitor and conserve insect biodiversity on a global scale [23,24]. In fact, metabarcoding of environmental samples generated by Malaise samples is quickly becoming the go-to method for biodiversity inventories worldwide [8,18,22]. However, one of the biggest advantages of metabarcoding—that it can be applied to hyperdiverse and poorly known communities—is also its main limitation. As Malaise sampling and metabarcoding are routinely applied to samples and regions unamenable to other methods of identification, we rarely know the *right* answer in terms of what species should occur where and what regional species pools we will be sampling. It is imperative that we test and scrutinize metabarcoding methods on well-known and comprehensively described taxa.

Lepidoptera, commonly known as butterflies and moths, provide a window of opportunity to validate the efficiency of mass sampling and metabarcoding methods and to assess their overall efficiency in recovering the local species pool. Lepidoptera are among the best-known and described insect orders worldwide (https://gbif.org), and thus the expected composition of regional species pools is unusually well established. At the same time, Lepidoptera are sometimes assumed to be poorly sampled by Malaise traps [21,25,26]—thus making a Lepidoptera-based assessment a conservative test case for the efficiency of high-throughput surveying.

For a test region, Sweden provides the ideal setting—since the lepidopteran fauna of Sweden and neighbouring countries is among the best explored in the world [27,28]. Catalysed by the activities of Linnaeus in the eighteenth century, the country has nurtured a long naturalist tradition, surviving until today and particularly noticeable in groups like Lepidoptera. In 2023, thousands of amateur and professional naturalists together reported an average of more than 120 occurrence records per species of Swedish Lepidoptera (https://gbif.org), at least an order of magnitude more than for any other group of insects. These naturalists sampled the national fauna by visual surveys and trapping methods, such as light traps, pheromone traps and bait traps.

Despite this long-lasting effort, the inventory of Swedish Lepidoptera is hardly complete [27]. New species are reported annually [29–31], whereas old ones are split into multiple taxa [32]. There may still be undiscovered, resident species, as well as cryptic taxa within ‘known’ taxa. Moreover, the fauna is far from static, and monitoring species’ ranges forms the basis of tracking regional change [33]. The ultimate efficiency and coverage of the combined enthusiast effort remain unvalidated, since the actual ground truth is unknown. An alternative approach—if it were powerful—would offer a rare opportunity to calibrate the naturalists’ efforts and bring us closer to the full picture of lepidopteran biodiversity.

We deployed Malaise traps across Sweden for a single year (2019), leveraging metabarcoding techniques to uncover new taxa and expand our understanding of species distributions, in a project known as the Insect Biome Atlas (IBA; https://insectbiomeatlas.org). Here, we look into what such a systematic survey can teach us about the country’s lepidopteran fauna. Firstly, our inquiry explores what proportion of the known Swedish Lepidoptera fauna was covered by the IBA survey. Subsequently, we explore how the IBA data compare to the data collected by naturalists using traditional methods. Finally, we look into the new discoveries generated by IBA: Swedish Lepidoptera species recorded in locations beyond known distribution ranges, species new to Sweden and putative species new to science.

## 2. Material and methods

### (a) Swedish species known to date

We retrieved the list of Lepidoptera records in the DynTaxa checklist [34] from the web interface at https://artfakta.se, together with the occurrence status of each species, as identified by staff at the Swedish Species Information Centre (Artdatabanken; https://artdatabanken.se; see data deposition info). DynTaxa [34] lists all species that have been recorded from the country by professional and amateur naturalists—documented in natural history collections, in published papers or in online databases—from the time of Linnaeus until the present.

### (b) Occurrence data collected by naturalists

All major sources of data on Swedish Lepidoptera known to us are available through the Global Biodiversity Information Facility (GBIF; https://gbif.org). To represent the knowledge accumulated by traditional means up until the completion of the IBA survey, we downloaded all Swedish lepidopteran occurrence data from GBIF for the time period up until and including 2019. We restricted our download to human observations, preserved specimens and the Artportalen dataset (Species Observation System of Artdatabanken, https://artportalen.se) as it represents the bulk of occurrence data in GBIF.

### (c) High-throughput biodiversity surveying

**Figure 1.**
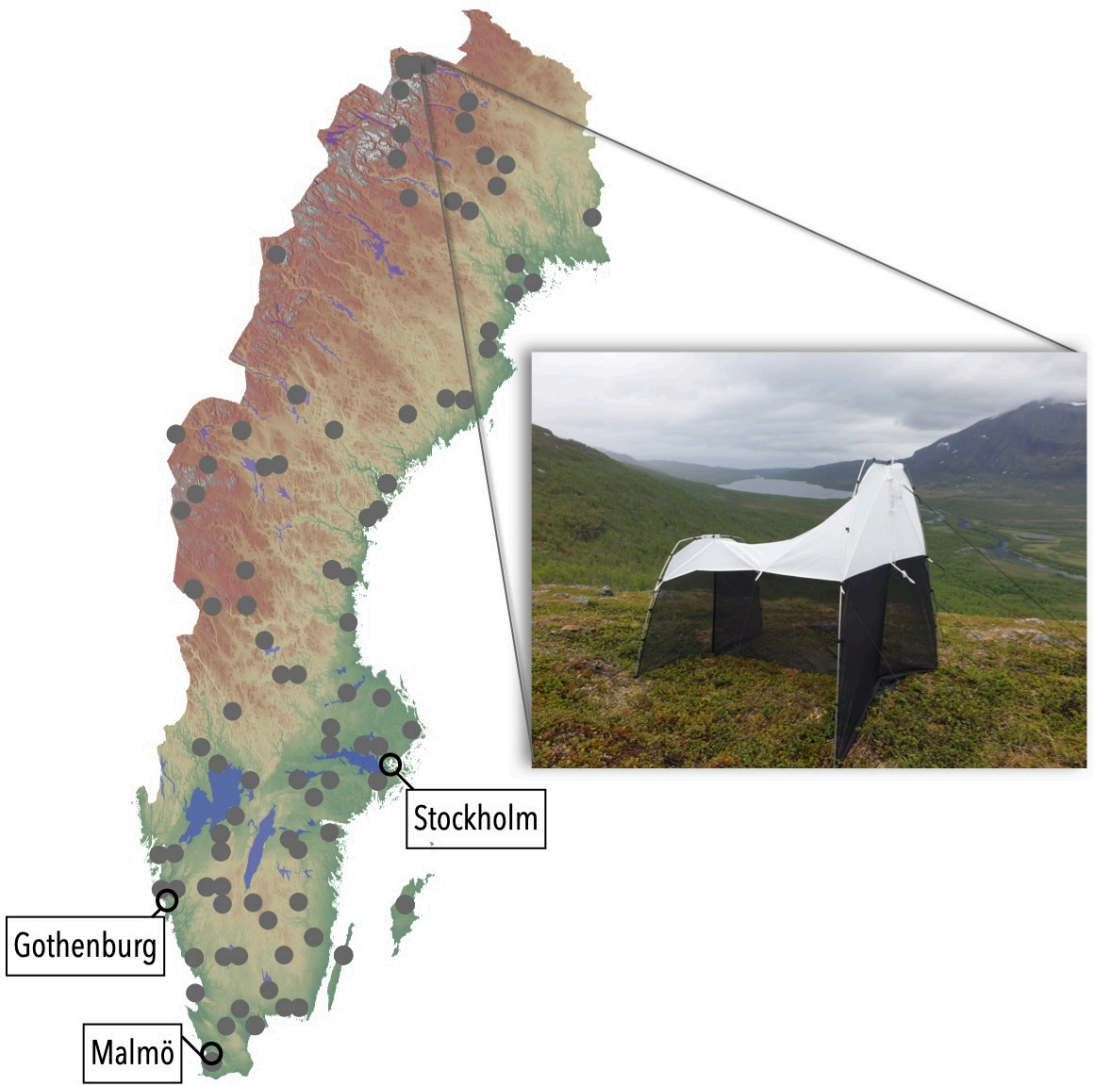
Distribution of IBA sampling sites in 2019 and main urban areas in Sweden with a photo showing one of the traps.

To generate records by high-throughput biodiversity surveying, we deployed Townes-type Malaise traps at 197 sites spread throughout Sweden (IBA survey; figure 1). Sites were selected in a systematic, hierarchical design, optimized to represent Swedish habitats and not to optimize the diversity or quantity of the insect catch. Traps were operated from January to December 2019 by citizen scientists, who replaced sample bottles weekly during spring to autumn and monthly or bi-weekly in winter if snow cover and wind permitted (October/November to March/April, depending on latitude). This yielded a total of approximately 4749 Malaise trap samples (see [35] for details).

All samples were metabarcoded according to the FAVIS protocol [36,37]. In short, we conducted a non-destructive mild lysis of the bulk sample, followed by DNA purification, preparation of amplicon libraries targeting a 418 bp fragment of the standard barcode region of the cytochrome *c* oxidase subunit I gene (COI) and sequencing the library pools on an Illumina NovaSeq 6000 platform using SPrime 500-cycle flow cells.

The resulting data were processed bioinformatically, as described by Miraldo *et al*. [37]. In short, after primer trimming, denoising and chimaera removal, all amplicon sequence variants (ASVs) were taxonomically annotated against a custom-made reference COI database (https://doi.org/10.17044/scilifelab.20514192.v4) using *SINTAX* [38] and then clustered using *SWARM* [39]. Clusters were then quality filtered by flagging noise (rare sequences) and likely nuclear DNA of mitochondrial origin (NUMTs) according to v. 1 of the filtering algorithm described in Miraldo *et al*. [35] and available at https://doi.org/10.17044/scilifelab.27202368.v1. Finally, taxonomic annotations were matched to the list of Swedish species, and mismatches were analysed in detail (see electronic supplementary material, methods S1). We analysed all clusters, including those flagged as potentially representing noise or NUMTs.

### (d) Evaluating the efficiency of the high-throughput survey

We focused on the following three aspects: (i) *species coverage*, that is, the proportion of known Swedish Lepidoptera species recovered, (ii) *comparison with naturalist data* with respect to taxonomic and regional coverage in the target year (2019), and (iii) *new discoveries*, specifically the detection of putative new species, new records for Sweden and records that significantly extended the known range of Swedish species. With respect to the third point, we note that the barcode reference libraries for Nordic Lepidoptera are nearly complete, thanks to Finnish, Norwegian, German and North American efforts, among others [28]. Thus, lepidopteran clusters not matching existing reference libraries but passing all quality filtering represent potentially known but not yet barcoded species or new, previously undescribed species (the former of which tend to be few in the Nordic fauna [28]).

### (e) Species coverage

We compared the list of species found in the IBA survey with the complete list of known Swedish lepidopteran species (DynTaxa) by matching species names. We did so for the entire fauna and at the family level. Here, we sorted families by the traditional but phylogenetically unsupported split into those with mostly small species (‘Microlepidoptera’) and those with mostly large species (‘Macrolepidoptera’; [40,41]; artfakta.se). To compare the taxonomic coverage of the IBA survey and naturalist-collected data in 1 year, we filtered GBIF observations to include only records from 2019. We then compared the number of occurrence records in the IBA data and in the GBIF dataset at the level of species and families.

### (f) Comparing regional coverage of naturalist and Insect Biome Atlas data in 2019

To compare the regional coverage of naturalist-collected and high-throughput survey data, we again used GBIF observations from 2019. We divided Sweden up into 125 roughly equally sized polygons using *R* package *simple features* [42,43] and then calculated the species richness of all lepidopterans, Macrolepidopterans and Microlepidopterans in each polygon for both naturalist-collected data and Malaise traps, whenever both data sources were available. We then calculated the proportion of the total occurrence records (i.e. the total number of unique species recorded in both datasets) recovered by each method to obtain a simple visual heuristic for comparison.

### (g) Validation of new discoveries

To analyse the quality of the metabarcoding data in generating new discoveries, we selected 20 samples for validation with full-length barcoding of individual specimens from the original samples. The identification of the strongest cases for potential new species and new records for Sweden was based on a manual analysis of IBA clusters that did not match the DynTaxa checklist (electronic supplementary material, results S1). To identify interesting new range records, we scanned the IBA data for species with at least one observation outside of their known range (according to GBIF data). For each lepidopteran species in GBIF, we calculated the observed range by computing the extent of occurrence (EOO) metrics via the minimum convex hull. We then calculated the longest distance between these ranges and the furthest record observed in the IBA data. We then selected 20 candidate species with the furthest distance from established EOOs, double-checking GBIF ranges against historical literature records [44]. We made the final selection of bulk samples for specimen sorting and full-length barcoding using an *R* script combining criteria (minimal set of samples containing at least 50 reads of 20 candidate species representing all three categories: new species, new country records and range expansions).

### (h) Individual-specimen barcoding

The 20 bulk samples selected were sorted into 35 taxon fractions following the Swedish Malaise Trap Project protocols [17]. We then searched the lepidopteran fraction for specimens matching the taxonomic identification of the candidate clusters. The selected specimens were processed with a rapid and efficient method of DNA barcoding described by Srivathsan *et al*. [45]. It combines non-destructive lysis, one-step polymerase chain reaction (PCR) amplification with indexed primers and MinION sequencing followed by barcode selection using ONTbarcoder software [46]. For these validations, we targeted the full 658 bp ‘Folmer region’ of the COI gene using LCO1490 and HCO2198 primers [47] with unique indices attached [46]. The resulting barcode sequences were then matched against the BOLD database [48] and BLASTed against GenBank data [49].

## 3. Results

The IBA high-throughput survey resulted in 544 263 ASVs grouped into 33 888 clusters (proxy for species), 1705 of which were annotated to Lepidoptera. The clustering of Lepidoptera sequences was highly consistent with species identifications based on traditional taxonomy; the errors, when they occurred, tended to be in the direction of splitting rather than lumping (precision 0.997, recall 0.980, homogeneity 0.997 and completeness 0.991 after noise filtering; [35]). Out of the 1705 Lepidoptera clusters, 1437 were successfully assigned to species using the default pipeline. This number increased to 1636 after the manual resolution of problems with taxonomic name conflicts and data quality problems in the BOLD reference library (electronic supplementary material, methods S1).

### (a) Species coverage

The known Swedish Lepidoptera fauna comprises 2990 species belonging to 76 families; 2697 of those are resident and regularly reproducing in the country (later referred to as *natural residents*). Some of the most species-rich families include the Tortricidae (leafroller moths) with over 400 species, Noctuidae (including Owlet moths) with around 350 species and Geometridae (including the Emeralds and Carpet moths) represented by about 320 species.

**Figure 2.**
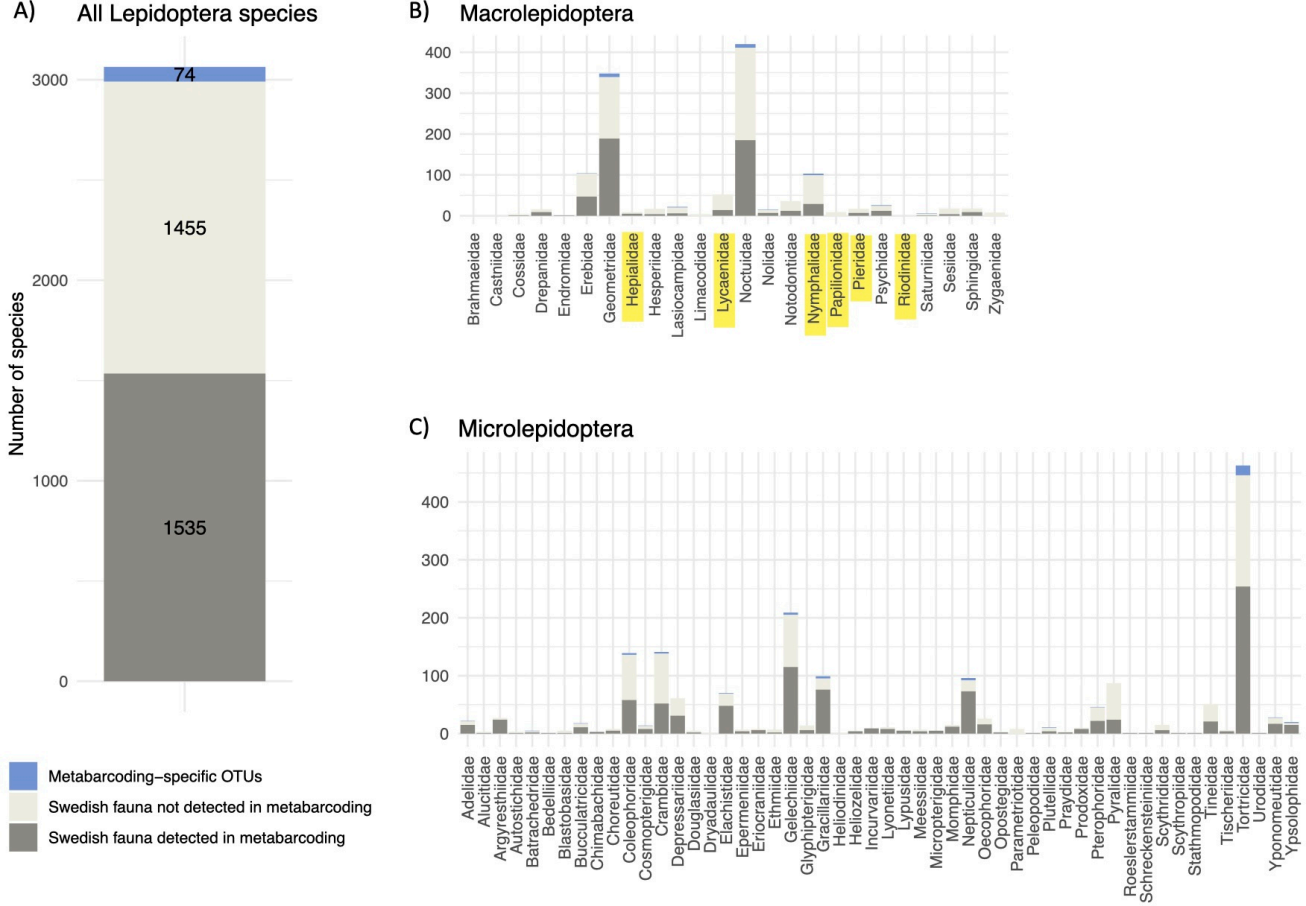
The proportion of the Swedish lepidopteran fauna detected by the high-throughput IBA survey using DNA metabarcoding of Malaise trap samples (A) across all taxa, (B) per Macrolepidopteran family and (C) per Microlepidopteran family. The dark grey sections of the bars correspond to Swedish species detected by the IBA survey; the light grey sections show species recorded from Sweden but not detected by IBA; and the blue sections show clusters (putative species) detected uniquely by IBA. Family names of butterflies are highlighted in yellow.

The IBA clusters matched 1535 species known from Sweden. Thus, a single year of high-throughput biodiversity survey recovered 51% of all lepidopteran species known from the country (figure 2A). All but three species were natural residents; the survey thus covered 1532 (57%) of the natural residents in the fauna.

The proportion of taxa recorded was slightly lower across families of Macrolepidoptera when compared with Microlepidoptera (44 versus 56%; electronic supplementary material, table S1) and tended to be even lower for butterflies (25%; electronic supplementary material, table S1; figure 2B,C, with butterfly families highlighted in yellow). Only nine of the 76 families were missing completely, all of them species poor. The missing species are associated with significantly fewer GBIF records than the ones recovered in the IBA survey (two-sided *t*‐test, *p* < 0.001), indicating that they are on average less common (electronic supplementary material, figure S1).

### (b) Comparison to data from naturalists

In 2019, naturalists reported 444 078 occurrence records of insects from Sweden; the Lepidoptera stood for 61% of those (272 380 records; electronic supplementary material, figure S2). The IBA data, viewed as species-space-time points with each point denoting the presence of a single cluster in a single sample, comprised 42 885 records, that is, 16% of the naturalist data for Lepidoptera. Lepidoptera was the only major insect group for which the naturalist data substantially exceeded that of the IBA survey viewed as simple occurrence records (electronic supplementary material, figure S2). The naturalist data from 2019 covered 2379 species of Lepidoptera, 1228 of which (52%) were also among the 1535 known species recovered in the IBA data (electronic supplementary material, figure S3).

**Figure 3.**
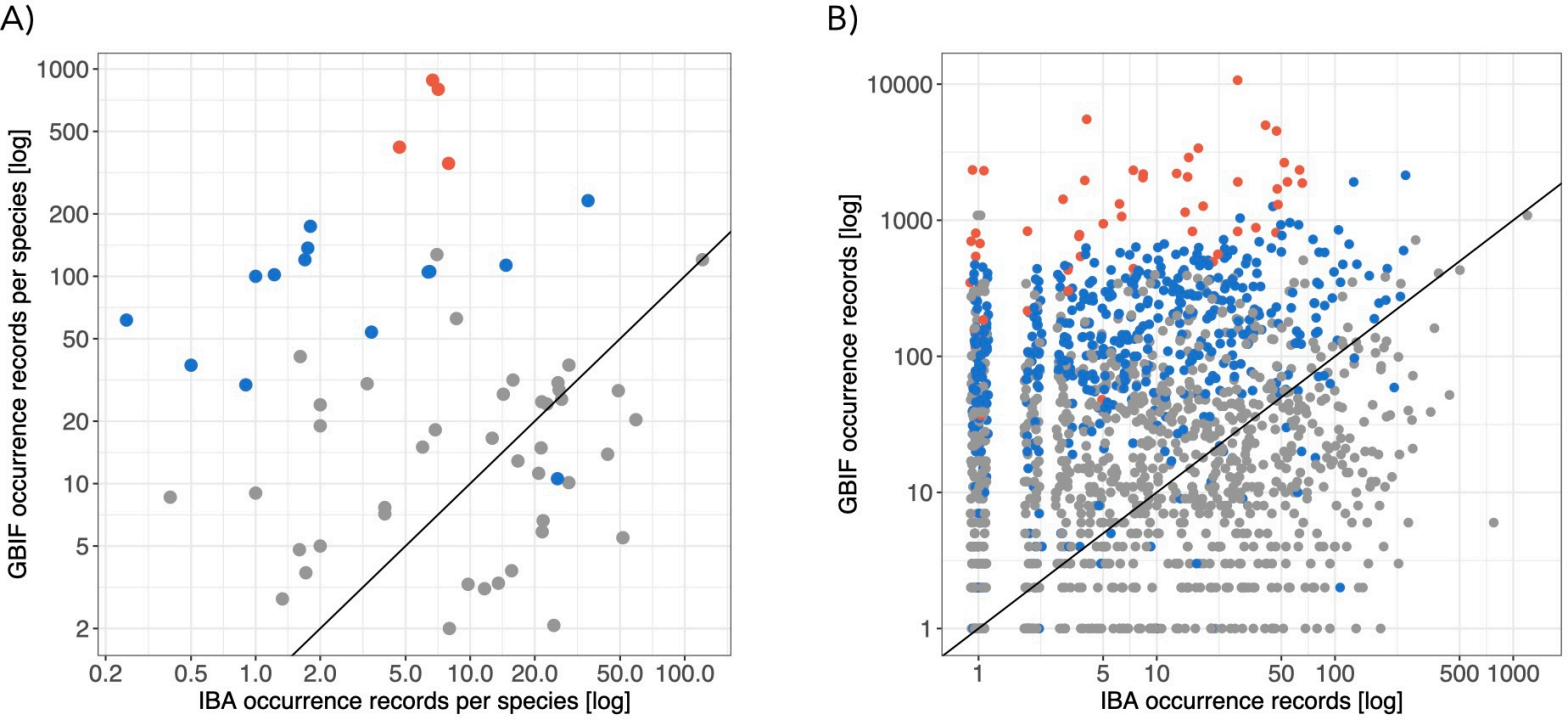
Comparison of the taxonomic coverage of naturalist (GBIF) and high-throughput survey (IBA) occurrence data in 2019. (A) Average number of occurrence records per species in each family; (B) number of occurrence records for each species (with jitter to improve visualization). Butterflies in red, other Macrolepidopterans in blue and Microlepidopterans in grey. For points above the line, there is more occurrence data from naturalists; below the line, there is more data from the high-throughput survey.

Taxonomically, the naturalist data are strongly biased towards families of butterflies and other Macrolepidopterans (figure 3A). The amount of IBA occurrence data equals or exceeds the naturalist data (in terms of the average number of records per known species) for around half of the families of Microlepidoptera (figure 3A) and for many individual species of Microlepidoptera (figure 3B). At the species level, there is a very weak correlation between the number of naturalist and high-throughput survey occurrence records (Pearson correlation coefficient (log scale) 0.11; figure 3B).

**Figure 4.**
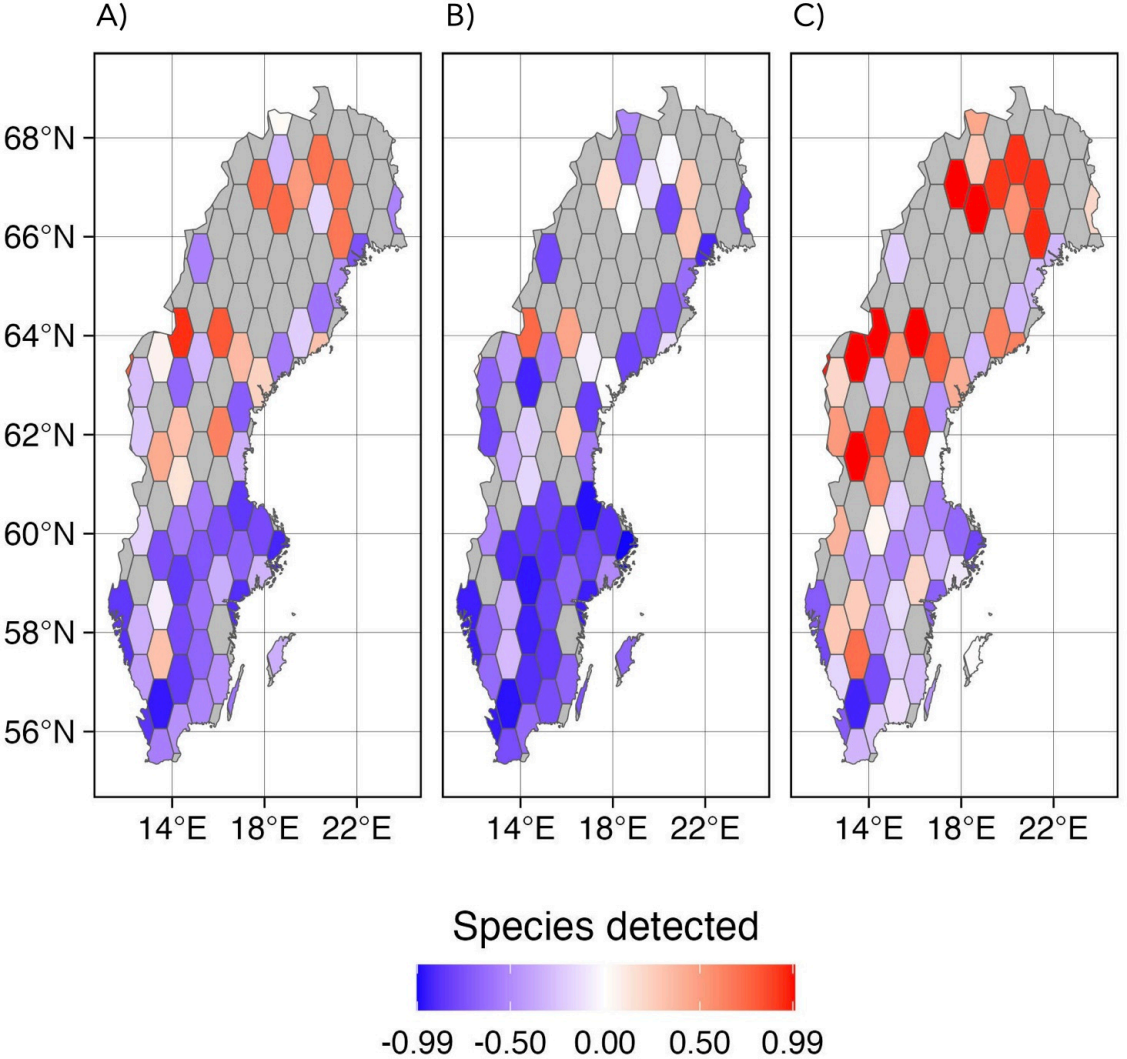
Comparison of the regional coverage of naturalist and high-throughput survey (IBA) data in 2019. (A) Difference in the proportions of species coverage in each spatial bin across all groups. (B) Ditto for Macrolepidopteran fauna. (C) Ditto for Microlepidopteran fauna. The colours reflect the difference in the proportion of species retrieved by the two surveys within each polygon. Blue colours represent more observations retrieved from naturalists (GBIF data), and red colours represent more data from the IBA survey. Grey polygons are areas not covered by the IBA survey.

Spatially, the amount of naturalist data largely appears to reflect human population density, with Stockholm, Mälardalen (lake area west of Stockholm), Gothenburg and Skåne (southern Sweden) standing out (figure 4A and electronic supplementary material, figure S4a). The naturalists excel at covering the species pools in these areas, particularly for butterflies and other Macrolepidoptera (figure 4B). The IBA data performed better than naturalists in recovering the local species pool in sparsely populated areas of the country. This is particularly noticeable for the Microlepidoptera (figure 4C).

### (c) Validation of new discoveries

The IBA data included 74 clusters that did not match any known Swedish species (figure 2A). Many of these belonged to small moth families (Microlepidoptera), but there were also potentially new clusters of Macrolepidoptera. In addition, the IBA data included several records that extended known species ranges considerably. Finally, there were 100 IBA clusters of Lepidoptera flagged by our bioinformatics pipeline as potential NUMTs or other noise.

After careful revision, we selected 20 clusters representing such new or surprising records for validation with full-length barcoding. We examined 12 clusters that did not match records in reference libraries and thus were potentially new to science (including six that had been flagged as noise). We also looked at four species not found in Sweden previously and four examples of conspicuous range expansions.

**Figure 5.**
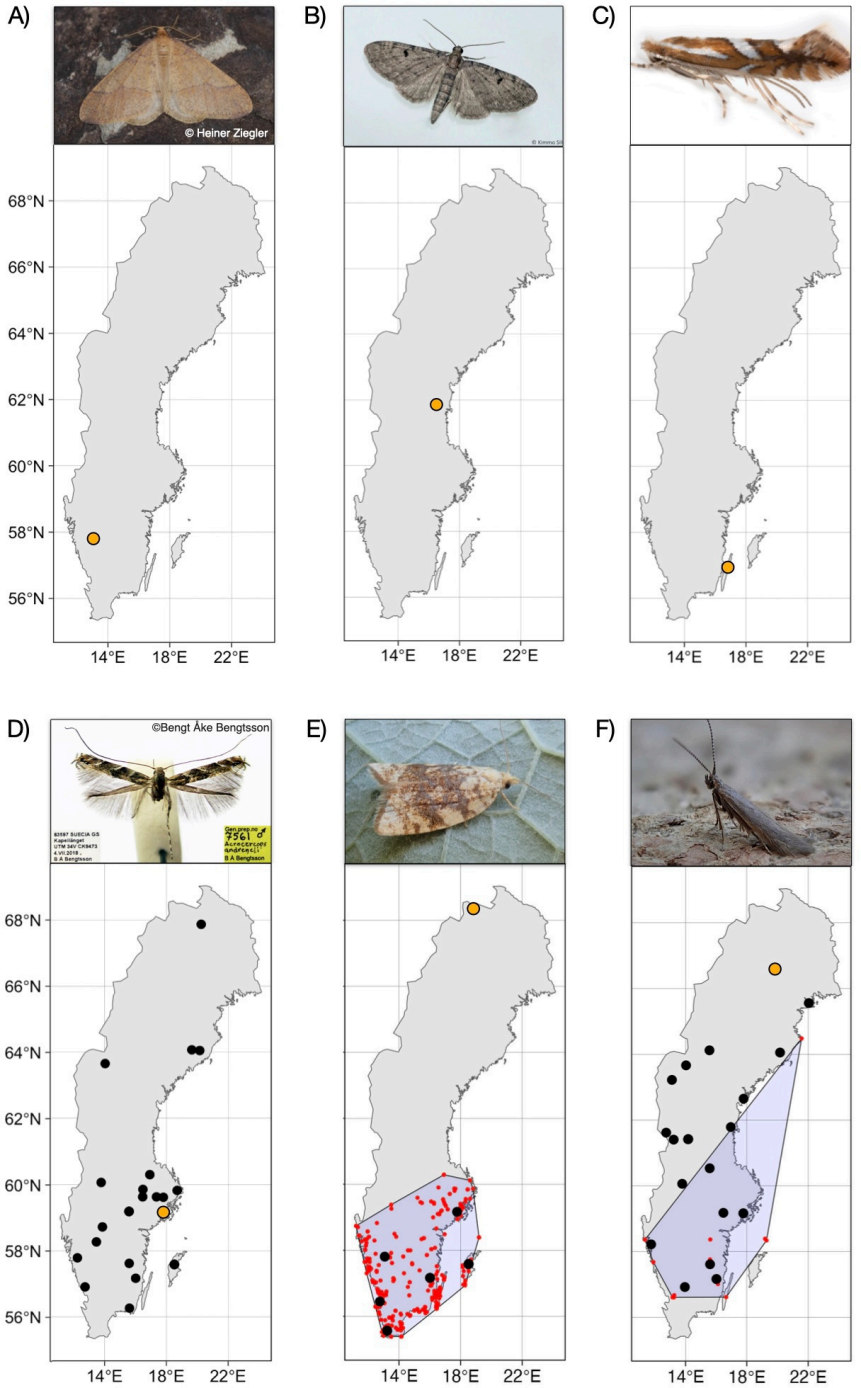
Examples of new discoveries resulting from the high-throughput IBA survey. Black dots indicate IBA occurrence records, and orange dots mark IBA samples that were sorted and individually barcoded. (A) *Agriopis budashkini* (Geometridae), a species described in 2009 from southeastern Europe, new to Sweden; (B) *Eupithecia groenblomi* (Geometridae) and (C) *Phyllonorycter mespilella* (Gracillariidae), both new to Sweden but known from neighbouring countries; (D) *Acrocercops andreneli* (Gracillariidae), a putative invasive species described in 2023; (E) *Aleimma loeflingiana* (Tortricidae) and (F) *Coleophora juncicolella* (Coleophoridae), both with known distribution significantly extended (previous records appear in red, surrounded by the minimal convex hull). Photo credits: A—© Heiner Ziegler—some rights reserved (CC BY NC), B—© Kimmo Silvonen (https://www.suomen-perhoset.fi) accessed 15 August 2024, C—© Chris Lewis (https://britishlepidoptera.weebly.com) accessed 22 October 2024, D—Bengt-Åke Bengtsson, E and F—Phil Barden (https://www.ukmoths.org.uk) accessed 15 August 2024.

We were able to validate three of the four species new to Sweden and two of the four range expansions—a third range expansion was also validated but turned out incorrect because our pipeline lumped two species and resolved the annotation as the more common southern species. Examples of the validated new discoveries are shown in figure 5 (see electronic supplementary material, results S1 and table S2 for details).

Eight clusters putatively representing new species, including three of six clusters flagged as noise and five of six flagged as authentic COI sequences, matched barcodes that were generated for individual specimens sorted out from Malaise traps (electronic supplementary material, table S2). The eight validated clusters represent genetically distinct COI variants, separated from known variants at distances suggesting they come from separate species. One of these variants apparently corresponds to *Acrocercops andreneli*, an invasive species that was not described until 2023 [50]. It was first reported from Sweden in 2024 [29], and the IBA data represent a unique record of its distribution in the country in 2019 (figure 5D).

## 4. Discussion

Malaise traps are frequently assumed to be poorly suited for sampling Lepidoptera, and DNA metabarcoding is dismissed as a surveying method due to the supposed poor data quality [17,25,26], however, other findings challenge these notions [51–53]. Similarly, an analysis of the Swedish Insect Inventory Project material using traditional morphological methods demonstrated that Malaise traps can be successful at recovering lepidopteran biodiversity [29]. Our findings corroborate the latter notion. The IBA survey sites and trap locations were not selected to optimize the quantity or diversity of the Lepidoptera catch. Nonetheless, we detected 57% of the natural resident species in a single year. Compared with traditional data, despite the popularity of Lepidoptera, the IBA survey provided better species and occurrence data coverage of sparsely populated regions, and of small and inconspicuous species. Further, the IBA data represent systematically collected sample-based data, which are considerably more powerful in documenting population densities and monitoring long-term trends than traditional data, mostly reported ad hoc. Validation of selected new discoveries with full-length individual-specimen barcoding also suggested that the data are reliable. Discoveries include seven putative new species, four species new to the country and two species occurring well beyond their known ranges. Below, we discuss each of these findings in turn.

The recovery of a large fraction of the lepidopteran fauna attests to two considerations: first, that Malaise traps offer an efficient method for sampling Lepidoptera, and second, that even a single year of high-throughput sampling is sufficient for characterizing a major part of insect biodiversity. Finding more than half of the nation’s lepidopteran diversity (1535/2990 species) in this single sampling effort should be put into perspective: first, our characterization of the national fauna was based on a mere 197 traps at 100 sites in a sparse grid (figure 1), and second, the point of comparison is centuries of tireless work by a large lepidopterist community. The 1535 species (not including new discoveries) detected in the IBA sampling bout compares even more favourably to the 2379 species recorded by the entire lepidopterist community of Sweden during 2019; it represents two-thirds of it. Importantly, the latter number draws on countless hours invested in all possible sampling methods from visual surveys to light traps, pheromone traps, bait traps and photography.

The proportion of the national species pool recorded by high-throughput sampling was distributed fairly evenly across lepidopteran families as well as between the traditionally recognized groups of Microlepidoptera versus Macrolepidoptera (figure 2). In other words, the constancy occurred irrespective of large variations in size, wing load, body plan, life history and diurnal activity. This is a hope-inspiring pattern, since it provides evidence against the notion that Malaise traps would disproportionately sample some taxa at the expense of others. Indeed, it elevates Malaise traps to a key method for sampling Lepidoptera. Admittedly, for certain exceptionally popular and charismatic groups, such as butterflies, traditional methods may still be more suitable, as our survey detected less than half of all butterfly species (figure 2). Likewise, few lepidopterists will be enthusiastic about the ethanol-soaked specimens generated by high-throughput sampling (but see [54] for instructions on how to make the most of this type of collection). Nonetheless, Malaise traps yield key metrics of the local lepidopteran fauna—with insights well beyond those provided by a traditional insect collection or photo collection.

While the data collected by lepidopterists tend to be biased towards large and conspicuous, preferably diurnal species occurring in densely populated areas of the country and popular summer destinations, the high-throughput survey data appear to provide a more even taxonomic and regional coverage (figures 3 and 4) Interestingly, the IBA data indicate that the species richness of local Lepidoptera communities (alpha-diversity) represents a high proportion of the regional species pool (gamma-diversity), resulting in low beta-diversity [55]. This suggests an interesting ecological pattern, i.e. low species turnover (i.e. low beta-diversity) of lepidopteran communities. This pattern contrasts with that of other taxa, such as plants and fungi [56], and reveals a key dimension of biodiversity patterns.

Metabarcoding is known to be associated with numerous data quality issues. Deviant sequences, which appear to represent distinct species, may instead reflect variation at the population level, heteroplasmy—the presence of several mitochondrial haplotypes within one individual [57–59]—or nuclear sequences of mitochondrial origin (NUMTs) [60]. Most NUMTs are short and include indels and/or premature stop codons (IPSCs) [61], which renders them easy to identify and filter out. However, some NUMTs do not have IPSCs and are therefore particularly difficult to distinguish from genuine COI sequences. This highlights the need for caution when unknown COI haplotypes are discovered in metabarcoding studies. In addition, contamination, chimaera formation during PCR [62] and sequencing errors can contribute to spurious clusters being detected in metabarcoding studies. The extensive congruence between the IBA survey and traditional Lepidoptera data, as well as the high success rate of the validation tests using full-length barcoding of individual specimens, suggest that our bioinformatic processing successfully tackled those issues, and the resulting data are reliable. Undoubtedly, this is partly explained by the completeness of the Lepidoptera reference library, which was used in quality filtering of the data, and the relatively long amplicon sequence targeted (418 bp), increasing the accuracy of library matching. Admittedly, data for insect clades less well represented in databases (i.e. Diptera, Hymenoptera—[27]) or with greater incidence of NUMTs (Orthoptera, Isoptera—[61]) could be less precise and likely more challenging to validate. A completely different type of concern is that variation in PCR primer sites may cause detection failure in DNA metabarcoding [63].

The validated new discoveries attest to the power of high-throughput surveying in providing novel information about species representing a cross-section of taxonomic and biological diversity (figure 5A–D). IBA detected three species new to Sweden. Among those, *Eupithecia groenblomi*, a small, scarce and hard-to-identify species in the family Geometridae, which was previously known from both Finland and Norway. Its host plant, European goldenrod (*Solidago virgaurea*), is widely distributed in Sweden. Thus, its previous absence from the Swedish checklist will likely reflect an oversight by Swedish lepidopterists. Another species, *Phyllonorycter mespilella,* is an inconspicuous member of the family Gracillariidae. It shares its food plant with several species that are extremely similar to one another and thus can be easily overlooked even by specialists. Molecular data, on the other hand, distinguish between those species with ease. Finally, *Agriopis budashkini* is a surprising find in Sweden, since its known range is Southern Europe, Balkans and Crimea. However, it is likely a cryptic species of which the entire range is unknown, since it is extremely easily confused with *Agriopis aurantiaria*. More speculatively, this cryptic species might have spread northwards already over a decade ago and be the hidden player behind reports of outbreaks of the geometrid moth known as *A. aurantiaria* in northern Norway [64]. An alternative explanation is that this observation results from hybridization or mitochondrial introgression from *A. budashkini* to *A. aurantiaria*, a phenomenon documented in several other species of European Lepidoptera [65]. It could also be a result of incomplete lineage sorting or phylogeographic processes—both of which can complicate species delimitation based on mitochondrial markers [66,67]. Those possibilities should be investigated further by specialists.

In terms of species-specific ranges, the high-throughput samples revealed records of species outside of their previously presumed ranges. The two validated range expansions represent slightly different cases. *Aleimma loeflingiana* (Tortricidae) is a leafroller moth that mainly feeds on oak [68]. Most of the IBA records match well the known distribution (GBIF records), but one record stands out. IBA recorded *A. loeflingiana* from the Torneträsk area, in far northern Sweden, way outside of its currently known range (figure 5E). This species is confined to oak, and despite the high latitude, oak does occur in the vicinity of the trap site; it is, therefore, possible that the species occurs there. However, the record could also represent a stray vagrant. The other validated example, *Coleophora juncicolella* (Coleophoridae), is a small case-bearing moth, which feeds on common heather (*Calluna vulgaris*) and bell heather (*Erica cinerea*). It is not recorded very commonly in GBIF and mainly from southern Sweden. The IBA data add many data points beyond the known species distribution, showing that *C. juncicolella* is in fact widespread throughout the country, as are the host plants (figure 5F). The species appears very likely to be a resident species overlooked in the northern part of its range.

Perhaps most impressively, we were able to validate the presence in Sweden of eight new COI variants, which were at least 3% distant from known COI sequences in existing reference libraries. Those new variants may represent previously unknown intraspecific variation within known Swedish species [69]. However, given the high coverage of lepidopteran taxa in reference libraries (41% of all insect species globally; boldsystems.org) and reliable bioinformatic clean-ups, this suggests that the new variants are reasonably likely to represent species new to science. A case supporting this conclusion is *A. andreneli*, detected as a putative new species in the IBA survey (2019) and described in 2023 [50]. Thus, surprisingly, less than 200 sampling locations from a nation the size of 450 000 km^2^ appears to be sufficient to discover a number of new species in one of the best-surveyed insect faunas of the world. As DNA-based approaches gain popularity, biodiversity discovery even within well-known habitats will become a common occurrence (i.e. [70]).

Overall, our findings point to the massive potential for insect biodiversity surveying and discovery through high-throughput methods, as well as to equally massive knowledge gaps among the few insect groups thought to be well known. To gain reliable knowledge of the fauna, we need to supplement the seminal collection of biological records by nature enthusiasts with systematic sampling designs generating reliable data in time and space. Needless to say, the two approaches should be seen as complementary rather than alternative to each other. Nonetheless, the massive insights generated by a single year of high-throughput sampling suggest that such methods should be added to any national tally of biodiversity. As a key bonus, these methods are based on *standardized sampling* with a *known sampling effort*, thus massively facilitating the downstream analysis of trends and drivers affecting ecosystems and the services they provide.

## Data Availability

Files with downloaded GBIF data are deposited on Figshare [71]. GitHub repository with data, code and scripts for the annotation analysis, the analysis of the GBIF data and for generating the figures in the paper [44]. Supplementary material is available online [72].
